# High Rate of Hypothyroidism in Multidrug-Resistant Tuberculosis Patients Co-Infected with HIV in Mumbai, India

**DOI:** 10.1371/journal.pone.0078313

**Published:** 2013-10-23

**Authors:** Aristomo Andries, Petros Isaakidis, Mrinalini Das, Samsuddin Khan, Roma Paryani, Chitranjan Desai, Alpa Dalal, Homa Mansoor, Reena Verma, Dolorosa Fernandes, Giovanni Sotgiu, Giovanni B. Migliori, Peter Saranchuk

**Affiliations:** 1 Médecins sans Frontières, Mumbai, India; 2 Sewri GTB Hospital, Chest Department, Mumbai, India; 3 Epidemiology and Medical Statistics Unit, Department of Biomedical Sciences, University of Sassari-Research, Medical Education and Professional Development Unit, AOU, Sassari, Sassari, Italy; 4 S. Maugeri Foundation, World Health Organization Collaborating Centre for Tuberculosis and Lung Diseases, Tradate, Italy; 5 Southern Africa Medical Unit (SAMU), Médecins Sans Frontières, Cape Town, South Africa; University of Michigan Medical School, United States of America

## Abstract

**Background:**

Adverse events (AEs) among HIV-infected patients with multidrug-resistant tuberculosis (MDR-TB) receiving anti-TB and antiretroviral treatments (ART) are under-researched and underreported. Hypothyroidism is a common AE associated with ethionamide, p-aminosalicylic acid (PAS), and stavudine. The aim of this study was to determine the frequency of and risk factors associated with hypothyroidism in HIV/MDR-TB co-infected patients.

**Methods:**

This was a prospective, observational cohort study, using routine laboratory data in a Médecins Sans Frontières (MSF) clinic in collaboration with Sewri TB Hospital, Mumbai, India. Hypothyroidism was defined as a thyroid stimulating hormone (TSH) result >10 mIU/L at least once during treatment. Patients having a baseline result and one additional result after 3 months were eligible for enrolment.

**Results:**

Between October 2006 and March 2013, 116 patients were enrolled, 69 of whom were included. The median (IQR) age was 38 years (34-43) and 61% were male. By March 2013, 37/69 (54%) had hypothyroidism after at least 90 days of treatment. Age, gender, CD4 counts and stavudine-based ART were not associated with the occurrence of hypothyroidism in multivariate models. The co-administration of PAS and ethionamide was found to double the risk of hypothyroidism (RR: 1.93, 95% CI: 1.06-3.54).

**Discussion:**

High rate of hypothyroidism was recorded in a Mumbai cohort of MDR-TB/HIV co-infected patients on treatment. This is a treatable and reversible AE, however, it may go undiagnosed in the absence of regular monitoring. Care providers should not wait for clinical symptoms, as this risks compromising treatment adherence. Simple, affordable and reliable point-of-care tools for measuring TSH are needed, especially in high MDR-TB burden countries. Our findings suggest the need for TSH screening at baseline, three months, six months, and every six months thereafter for HIV-infected patients on MDR-TB treatment regimens containing PAS and/or ethionamide, until newer, safer and more efficacious MDR-TB regimens become available.

## Introduction

The number of people being initiated on treatment for multidrug-resistant tuberculosis (MDR-TB) almost doubled between 2009 and 2011 as a result of steady annual increases in 12 of 27 countries having a high MDR-TB burden. This includes India where 3384 cases were enrolled on MDR-TB treatment in 2011 compared to 1136 in 2009. Improved capacity for MDR-TB case detection has also contributed to the increasing number of cases[1]. The increasing trend towards drug-resistant TB in several high burden countries throughout the world will pose a serious public health threat for future generations[1-3].

MDR-TB is defined as tuberculosis that is resistant to isoniazid and rifampicin, the two most effective anti-TB drugs in the first-line regimen[2,4]. Treatment for patients having MDR strains requires use of second-line anti-TB drugs and a treatment duration of at least 20 months[2,4-6]. An MDR-TB regimen is best chosen based on drug susceptibility testing (DST) results and assessment of the past treatment history, and typically consists of a minimum of 5 drugs, of which 4 are second-line anti-TB drugs: any first-line drugs thought to be effective, one injectable agent, one fluoroquinolone, and more than one of the oral bacteriostatic second-line anti-TB drugs (cycloserine/terizidone, ethionamide/prothionamide, +/- p-aminosalicylic acid or PAS)[6,7]. In cases having more advanced resistance, such as extensively drug-resistant TB (XDR-TB), all oral bacteriostatic second-line anti-TB drugs may need to be used[8,9], as well as some ‘group 5’ drugs having unclear efficacy (clofazimine, linezolid, etc)[5,10,11].

Second-line anti-TB drugs are more likely to cause adverse events (AE) than the first-line anti-tuberculosis drugs used in drug-sensitive TB treatment[2,12,13]. One of the known AEs related to MDR-TB treatment is hypothyroidism. The second-line anti-TB drugs associated with hypothyroidism include ethionamide, prothionamide, and PAS[2,4,14]. These drugs can cause hypothyroidism by inhibiting thyroid hormone synthesis through a mechanism of iodine organification inhibition[15,16]. Clinical hypothyroidism presents with the following symptoms and signs: slowing in both mental and physical activities, dry skin, cold sensitivity, fatigue, muscle cramps, voice changes, and constipation[17]. In rare cases, hypothyroidism can manifest severely as cardiac disease and development of a pericardial effusion[18].

MDR-TB treatment in patients co-infected with HIV remains a challenge. Patients are required to take a large number of medicines for treating both diseases and additionally, for managing AEs. Antiretrovirals and drug-resistant TB drugs have potentially overlapping AEs[14,19,20]. In a multivariate analysis of a study of HIV patients receiving ART, cumulative dosing of stavudine was associated with subclinical hypothyroidism[21]. Efavirenz, amprenavir, lopinavir, and ritonavir were also found to be associated with hypothyroidism[22]. A number of studies looking at hypothyroidism in MDR-TB cohorts not co-infected with HIV and mixed cohorts of HIV/MDR-TB co-infected and non-HIV co-infected patients have identified ethionamide and PAS as the most likely culprit drugs[23-26]. However, there are limited data on hypothyroidism in MDR-TB patients co-infected with HIV.

The aim of this study was to determine the frequency of and risk factors associated with hypothyroidism in a cohort of MDR-TB patients co-infected with HIV in Mumbai, India.

## Methods

### Study design

The study was a prospective, observational cohort study using routine clinical and laboratory data. 

### Setting and study population

Médecins Sans Frontières (MSF) started an HIV project in Mumbai, India in 2006, into which treatment for both drug-sensitive and drug-resistant TB were integrated. HIV-infected patients initiated on a multidrug-resistant TB regimen between October 2006 and March 2013 were eligible for enrolment in this study.

### Treatment protocol and follow-up

All patients received therapy for drug-resistant TB (DR-TB) using a model of care that involves ambulatory, community-based, directly observed treatment (DOT) as previously described elsewhere[19]. An individualized treatment regimen was designed for each patient based on first- and second-line DST results and the patient’s previous TB treatment history. Any patient who required immediate treatment was initiated on an empiric treatment regimen consisting of pyrazinamide, capreomycin, moxifloxacin, ethionamide, cycloserine, and PAS while awaiting the DST result; treatment was then either individualized once DST results became available, or the empiric regimen continued for culture-negative patients with a strong suspicion of drug-resistant TB based on TB treatment history. Some patients initially enrolled in the program were treated for a duration of 18 months following the WHO guidelines at that time[4]. Once the WHO guidelines were updated in 2011, the treatment duration was adjusted accordingly to a minimum of 20 months[6]. Patients underwent monthly follow-up in the MSF clinic and thyroid-stimulating hormone (TSH) levels were tested every 3 months. When patients required hospitalization, MSF collaborated with private and government hospitals; Sewri TB Hospital was the only government hospital in Mumbai having hospital wards designed to accommodate tuberculosis patients.

Patients diagnosed with TB were eligible for antiretroviral therapy (ART) irrespective of the CD4 count. Thus, all patients not already on ART when they enrolled in the MSF clinic received ART as soon as they tolerated second-line TB treatment. Patients received two nucleoside/tide reverse transcriptase inhibitors (NRTIs) and one non-nucleoside reverse transcriptase inhibitor (NNRTI) in their first-line ART regimen. Protease inhibitor-based regimens were used for patients in need of second-line and third-line ART.

TSH was measured using the serum chemiluminescence method in a private laboratory (RLS, Religare, Mumbai, India) accredited by the College of American Pathologists. The reference range of laboratory detection for TSH was 0.35-5.5 mIU/L.

### Data collection and statistical analysis

Data related to HIV/MDR-TB co-infected patients initiated on treatment between October 2006 and March 2013 were included in the analysis. Data were routinely collected during each consultation and entered into handwritten patient files and an electronic database. Both HIV and TB data were collected from integrated patient file systems. 

Hypothyroidism can be differentiated into subclinical (defined as elevated serum TSH above the upper limit in combination with normal free thyroxine (T4)) versus overt hypothyroidism (elevated TSH, usually above 10 mIU/L, in combination with low T4)[17]. In this study, hypothyroidism was defined as at least one TSH value > 10 mIU/L after at least 3 months of second line TB treatment.

Patient characteristics were summarized using descriptive statistics. We used t-test, chi-square or Fisher’s exact test to assess differences of variables between groups, as appropriate. The time-to-hypothyroidism was measured using Kaplan-Meier analysis (data were censored by 30^th^ March 2013). We performed bi- and multi-variate analyses to assess risk factors associated with the occurrence of hypothyroidism. We calculated Relative Risks and adjusted Hazard Ratios (with 95% Confidence Intervals) using bivariate and Cox regression models respectively. Factors used as predictors included age, sex, baseline CD4 count, baseline body mass index (BMI), tuberculosis site (pulmonary vs. extrapulmonary), and TB treatment regimen. We used the cohort’s median age as a cut-off point in the modeling. When patients had both pulmonary and extrapulmonary TB, we classified them as extrapulmonary TB for the modeling. A p-value of less than 0.05 was considered to indicate statistical significance. SPSS (IBM, SPSS, Statistics, version 20) was used for analysis.

### Ethics

The study satisfied the criteria for reports using routinely collected programmatic data, set by the Médecins Sans Frontières independent Ethics Review Board (MSF ERB) in Geneva, Switzerland. Informed consent to the patient was not obtained since the data used in the study were routinely collected for treatment monitoring. The MSF ERB specifically approved the study and waived the need for consent. 

## Results

**Figure 1:**
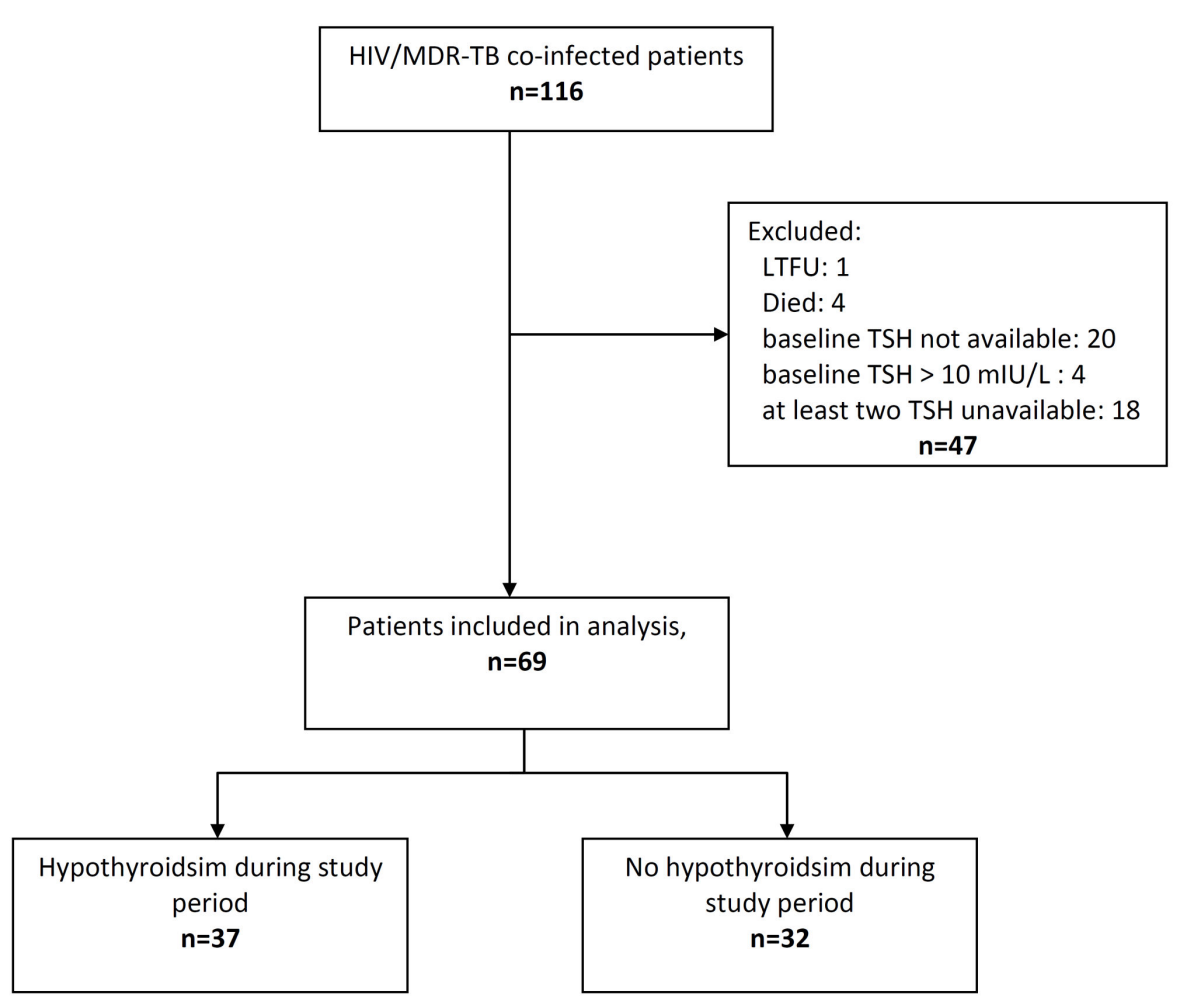
HIV/MDR-TB co-infected patients who developed hypothyroidism during the study period.

Between October 2006 and March 2013, 116 drug-resistant TB patients co-infected with HIV were enrolled in the MSF clinic, of whom ninety-six (82.8%) had a baseline TSH value measured. Twenty-three of these (24.0%) did not fulfill the study definition and were excluded from the analysis; 18 patients have no follow up TSH measurement after 3 months, 1 patient lost to follow up, and 4 patients died before TSH follow up. Another four patients (4.2%) had an elevated TSH at baseline and were also not included. Thus, a total of sixty-nine patients met the study criteria and were included in the analysis, as shown in Figure 1. Clinical characteristics of these patients are presented in Table 1.

**Table 1:** Bi-variate analysis for development of hypothyroidism during treatment among HIV/MDR-TB co-infected patients, Mumbai, India, 2013.

**Explanatory Variable**	**Total (n=69) n (%)**	**Patients developed hypothyroidism n (%)**	**Patients did not develop hypothyroidism n (%)**	**p-value**	**RR (95% CI)**
**Age** (years, mean ± SD)	37.38 ± 9.61	38.84 ± 9.11	35.69 ± 10.04	0.176	-
**Sex of patients**					
Male	42 (60.9)	22 (59.5)	20 (62.5)	0.796	0.94 (0.60-1.47)
Female	27 (39.1)	15 (40.5)	12 (37.5)		
**Category of TB**					
Pulmonary	41 (59.4)	21 (56.8)	20 (62.5)	0.628	0.90 (0.58-1.39)
Extra-pulmonary	28 (40.6)	16 (43.2)	12 (37.5)		
**Baseline CD4 count**					
Less than 200	49 (71.0)	30 (81.1)	19 (59.4)	0.047*	1.75 (0.92-3.31)
200 and above	20 (29.0)	7 (18.9)	13 (40.6)		
**ART regimen**					
Stavudine	15 (21.7)	7 (18.9)	8 (25.0)	0.541	0.84 (0.46-1.52)
No stavudine	54 (78.3)	30 (81.1)	24 (75.0)		
**Medication on treatment**					
Eto and PAS	24 (34.8)	8 (21.6)	16 (50.0)	0.014*	1.93 (1.06-3.54)
Other than Eto/PAS combination	45 (65.2)	29 (78.4)	16 (50.0)		
**Baseline BMI** (mean ± SD)	17.37 ± 4.05	17.83 ± 4.09	16.83 ± 4.00	0.310	-
**Total number of patients (N**)	**69**	**37**	**32**		**69**

Eto: Ethionamide, PAS: Para-Aminosalicylic acid, RR: Relative Risk, CI: Confidence Intervals

* Levels of significance are p < 0.05

**Figure 2:**
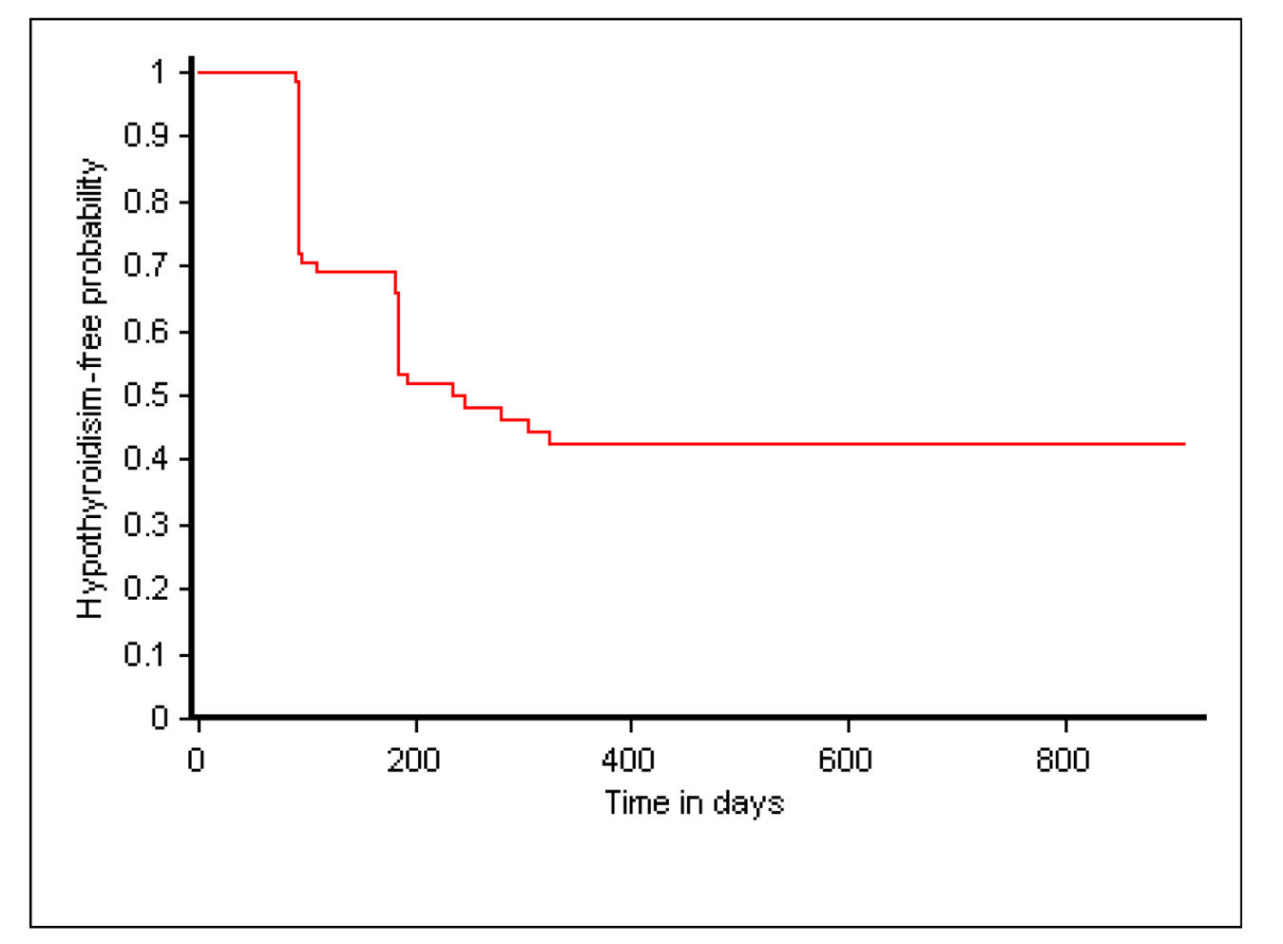
Time-to-Hypothyroidism following initiation of MDR-TB treatment using Kaplan-Meier analysis.

Out of 69 patients analyzed as of March 2013, 37 (53.6%) developed hypothyroidism during the study period (Figure 1). Twenty-three of them (62%) were diagnosed with hypothyroidism at 90 days (i.e. the first TSH follow up), while 12 more patients were diagnosed at 180 days. The remaining two patients were diagnosed at 240 and 300 days respectively. Figure 2 shows the Kaplan-Meier curve of time-to-hypothyroidism after MDR-TB treatment initiation.

In 67 out of 69 patients included in the analysis, ethionamide and/or PAS were included in the regimen. Forty-five (67%) patients received both ethionamide and PAS, while the remaining 22 patients received either ethionamide or PAS. There was no difference in the time-to-hypothyroidism between the patients who received both drugs and the ones who received just one of the two. There was a significant association between the use of both ethionamide and PAS in the regimen and developing hypothyroidism (p = 0.014) as presented in Table 1. Co-administration of these two agents increased the risk of hypothyroidism by two-fold (relative risk: 1.93, 95% CIs: 1.06-3.54). Age, sex, category of TB (pulmonary or extra-pulmonary) and stavudine were not associated with the occurrence of hypothyroidism. No factors were found to be associated with the occurrence of hypothyroidism in multivariate models (Table 2).

**Table 2:** Risk factor analysis for development of hypothyroidism during treatment among HIV/MDR-TB co-infected patients, Mumbai, India, 2013.

**Explanatory Variable**	**Patients developed hypothyroidism n (%)**	**Patients did not develop hypothyroidism n (%)**	**Adjusted hazard ratios (95% CI)**
**Age group of patients**			
Less than 38 years	18 (48.6)	15 (46.9)	1.04 (0.51-2.13)
Equal or more than 38 years	19 (51.4)	17 (53.1)	
**Sex of patients**			
Male	22 (59.5)	20 (62.5)	0.93 (0.47-1.86)
Female	15 (40.5)	12 (37.5)	
**Category of TB**			
Pulmonary	21 (56.8)	20 (62.5)	1.02 (0.51-2.06)
Extra-pulmonary	16 (43.2)	12 (37.5)	
**Baseline CD4 count**			
Less than 200 cells/µl	30 (81.1)	19 (59.4)	2.05 (0.86-4.89)
200 and above	7 (18.9)	13 (40.6)	
**ART regimen**			
Stavudine	7 (18.9)	8 (25.0)	0.85 (0.35-2.05)
No stavudine	30 (81.1)	24 (75.0)	
**Medication on treatment**			
Both Eto and PAS	8 (21.6)	16 (50.0)	1.84 (0.80-4.24)
Other than Eto/PAS combination	29 (78.4)	16 (50.0)	

(n=69). Eto: Ethionamide, PAS: Para-Aminosalicylic acid, CI: Confidence Intervals

## Discussion

Hypothyroidism was recorded in the majority (53.6%) of this Mumbai cohort of HIV/MDR-TB co-infected patients on both antiretroviral and second-line TB treament. In a previous analysis of data from this cohort we have found lower rate of hypothyroidism (32%)[14]. We assume that the increased rate is due to longer exposure of the cohort to 2^nd^ line drugs and a greater proportion of patients receiving both PAS and ethionamide. All 37 patients in whom hypothyroidism was detected were asymptomatic. An MSF clinic protocol assured that the patients who have elevated TSH > 10 mIU/L, whether symptomatic or not, would receive levothyroxine 50-150 μg daily based on the patient’s weight and titrated according to the response to therapy. This approach prevents the emergence of symptomatic hypothyroidism in patients attending the MSF clinic.

Although stavudine has been found elsewhere to be associated with the occurrence of hypothyroidism[21], no association was found in this study. It should be noted that stavudine is no longer preferred in a first-line ART regimen[27]; only 15/69 (21.7%) patients were still on a stavudine-based ART regimen during the study period, of which seven patients developed hypothyroidism. Similarly sex was previously found to be associated with hypothyroidism in HIV-infected patients but we found no such association in our Mumbai cohort of co-infected patients on treatment[21].

In our study, lower CD4 cell counts were associated with hypothyroidism in bivariate analysis, which may be a result of HIV infection itself being associated with thyroid dysfunction. Patients with low CD4 cell counts may be at higher risk of opportunistic infections that could affect the thyroid, such as hypothyroidism due to infiltration of Kaposi’s Sarcoma cells into the thyroid gland[28]. According to the literature, thyroid dysfunction may have infectious, hemorrhagic, or neoplastic etiologies[29-31].

The mechanism of hypothyroidism due to second-line anti-TB drugs is clear and it is both treatable and reversible (upon discontinuation of the offending drug)[15,16]. Even mild or subclinical hypothyroidism should not be overlooked as it may increase the risk of co-morbidities such as depression[32]. In addition to a potential negative effect on adherence to MDR-TB treatment, untreated depression will also result in suboptimal adherence to antiretroviral treatment[33]. In our setting, we managed to prevent overt hypothyroidism by detecting it early and treating it promptly.

A high rate of hypothyroidism (69%) was also found in a DR-TB cohort from Lesotho having an HIV co-infection rate of almost 70%[24]. However, findings have varied in other studies: a study of MDR-TB adverse events in Tomsk, Russia reported a 17.2% incidence of hypothyroidism[34], while five out of seven patients (71.4%) treated for MDR-TB with both PAS and prothionamide in the United Kingdom also developed hypothyroidism. Though the number of patients in the latter study was small, it was the largest cohort of patients developing drug-induced hypothyroidism in the United Kingdom[23]. Co-infection with HIV was not discussed in either of these studies. Another study conducted in KwaZulu-Natal province in South Africa looking for adverse events in an MDR-TB program reported hypothyroidism in 36%, the most common adverse event among the cohort. Most of the patients enrolled in this study (81%) were HIV-positive and receiving ART. [35]

In Sewri TB hospital, where the largest cohort of MDR-TB patients is being treated in Mumbai, unpublished data related to 115 patients on MDR-TB treatment between the years 2005 and 2010 showed only six patients (5%) had hypothyroidism. However, these six patients had TSH laboratory testing only when they were symptomatic, as recommended in the Indian national guidelines[36]; three of the patients had thyroid swelling and the other three had symptoms of hypothyroidism. Since TSH was not systematically tested in all the MDR-TB patients and had to be paid for by patients, the true rate of hypothyroidism is likely to be very much underestimated.

Despite being one of the largest cohorts of HIV/MDR-TB patients in India, the relatively small number of patients in this report is one of the study limitations. On the other hand, these operational data reflect the reality of patient care in our setting, which has room for improvement. Of the 47 patients excluded from the study (Figure 1), 18 of them did not have two TSH measurements (i.e. at baseline and 3 months after TB treatment started), 1 patient was lost to follow up, 4 patients died before TSH follow up, 4 patients have elevated TSH at baseline, and 20 of the patients did not have a baseline TSH result. The study also did not measure the prevalence of iodine deficiency, although it should be noted that India is among those countries where iodine intake tends to be adequate[37].

In India and elsewhere, ethionamide is included in the national standardized MDR-TB regimen. PAS is also used if other second-line agents such as ethionamide and/or cycloserine cannot be used. The high rate of hypothyroidism found in this study suggests the need for routine screening for hypothyroidism in HIV/DR-TB co-infected patients taking ethionamide and/or PAS. This is currently not the case in India and other resource-limited settings, where protocols recommend measurement of TSH only if clinically indicated, and not as part of routine monitoring[36]. WHO guidelines for programmatic management of drug-resistant tuberculosis recommend TSH monitoring at least every 6 months while other guidelines advise screening every three months[2,4]. We suggest TSH screening be done at baseline, at three months, at six months, and every six months thereafter for HIV-infected patients on MDR-TB treatment regimens containing PAS and/or ethionamide, based on our findings that most of the patients developed hypothyroidism within 90 days.

TSH testing can be challenging in resource-limited settings due to limited availability. In Sewri TB hospital, a public facility, patients with MDR-TB have no access to TSH testing unless symptomatic (and often at a fee). A point-of-care (POC) diagnostic tool for TSH screening would certainly be useful in such settings. One such tool, TSH-CHECK-1, has shown promising results as a screening test[38]; however, confirmatory TSH testing would still be necessary following any positive TSH-CHECK-1 result, since this rapid test does not have sufficient specificity.

Ongoing efforts to find a better, more efficacious and less toxic second-line TB regimen requiring considerably shorter treatment duration should be the priority of the TB community. Until that happens, we need to reduce as much as possible the frequency of serious adverse events in MDR-TB patients with or without HIV co-infection by systematically screening to detect any adverse events early, including hypothyroidism.
